# Enhanced Stability of Gold Magnetic Nanoparticles with Poly(4-styrenesulfonic acid-co-maleic acid): Tailored Optical Properties for Protein Detection

**DOI:** 10.1186/s11671-017-2303-6

**Published:** 2017-09-25

**Authors:** Xiaomei Zhang, Qinlu Zhang, Ting Ma, Qian Liu, Songdi Wu, Kai Hua, Chao Zhang, Mingwei Chen, Yali Cui

**Affiliations:** 1 College of Life Sciences, Northwest University, Xi’an, 710069 China; 2 School of Medicine, The First Affiliated Hospital of Xi’an Jiaotong University, Xi’an, 710061 China; 3 College of Agricultural, Henan University of Science and Technology, Luoyang, 471003 China; 4 No. 1 Hospital of Xi’an City, Xi’an, 710068 China

**Keywords:** Poly(4-styrenesulfonic acid-co-maleic acid) sodium salt, Gold magnetic nanoparticles, Dispersity, Conjugation, Protein detection

## Abstract

Gold magnetic nanoparticles (GoldMag) have attracted great attention due to their unique physical and chemical performances combining those of individual Fe_3_O_4_ and Au nanoparticles. Coating GoldMag with polymers not only increases the stability of the composite particles suspended in buffer but also plays a key role for establishing point-of-care optical tests for clinically relevant biomolecules. In the present paper, poly(4-styrenesulfonic acid-co-maleic acid) (PSS-MA), a negatively charged polyelectrolyte with both sulfonate and carboxylate anionic groups, was used to coat the positively charged GoldMag (30 nm) surface. The PSS-MA-coated GoldMag complex has a stable plasmon resonance adsorption peak at 544 nm. A pair of anti-D-dimer antibodies has been coupled on this GoldMag composite nanoparticle surface, and a target protein, D-dimer was detected, in the range of 0.3–6 μg/mL. The shift of the characteristic peak, caused by the assembly of GoldMag due to the formation of D-dimer-antibody sandwich bridges, allowed the detection.

## Background

Taking advantage of a specific magnetization property, i.e., superparamagnetism, magnetic nanoparticles have been widely investigated for biomedical applications, such as drug/gene delivery, magnetic resonance imaging, and biological assays [1–3]. Gold magnetic nanoparticles (GoldMag), composed of Fe_3_O_4_/Au, not only have the physicochemical properties of iron oxide nanoparticles but also present gold nanoparticles characteristics such as easy surface-functionalization and unique optical properties. These distinguishing features have attracted great attention in the field of biology [4, 5], especially in biomolecule detection based on optical properties. As an example, Wang et al. used Fe_3_O_4_-Au rods as optical probes for multiplex pathogen detection [6]. However, like with other nanoparticles, the high surface energy forces the GoldMag particles toward to each other so that they form clusters in buffer solution. This greatly limits their application for optical detection in the biomedical area. It is therefore crucial to prevent the aggregation of GoldMag and to ensure a stable colloid solution. Customizing GoldMag for detecting target molecules based on coating with, polymers has a high utility. It has been reported that the dispersity of GoldMag can improved through surface modification with various macromolecular organic compounds, such as 11-mercaptoundecanoic acid (MUA) [7], polystyrenesulfonate (PSS) [8], and polyethylenimine (PEI) [9]. MUA was brought on the surface of GoldMag using the ligand-exchange strategy. It enhanced the stability of GoldMag colloidal solutions through surface modification with MUA [7]. These MUA-GoldMag have been used in protein detection based on optical properties. However, the chain of MUA was too short to provide enough steric hindrance for assuring sufficient dispersity of particles. Moreover, the low density of carboxyl groups in MUA limits the amount of protein that can be absorbed to the GoldMag. These disadvantages restrict the application of MUA-particles in optical instruments and limit the sensitivity required for the detection of biomarkers.

Poly(4-styrenesulfonic acid-co-maleic acid) (PSS-MA) (PSS-MA 3: 1, Mw ~ 20,000), a block copolymer, is made by the covalent bonding of PSS and polymaleic acid and contains both sulfonate and carboxylate groups. The electrostatic repulsion provided by the great number of carboxyl groups and sulfonate groups, as well as the steric hindrance coming from the long polymer chain of PSS-MA make this polymer very useful in maintaining the stability of the nanoparticles. In fact, PSS-MA has been used as a stabilizer in the preparation of nanoparticles, such as iron oxide nanomaterials, palladium and Ag-Au bimetallic nanostructures [10–12]. Johnston et al. reported that copolymer coated iron oxide nanoclusters assure a higher degree of electrosteric stabilization than single macromolecule polymer-coated iron oxide nanoclusters [13]. In addition, the great number of carboxyl groups in the polymaleic acid moiety of PSS-MA allows chemical modification with biomolecules for biomedical applications.

D-dimers are stable end products of the degradation of cross-linked fibrin, resulting from enhanced fibrin formation and fibrinolysis [14]. Determination of D-dimer level in blood is widely used in diagnosis of thromboembolic events and myocardial infarction [15, 16]. Here, we used D-dimer as a model to evaluate the potential of using PSS-MA-GoldMag for optical detection of specific proteins. We used a pair of anti D-dimer antibodies for immobilization on PSS-MA-GoldMag in order to form probes for the detection of D-dimer by the double antibody sandwich immunoassay.

Thus, in the present study, PSS-MA was used for surface modification of GoldMag. This not only enhanced the stability of the magnetic nanoparticles but also mediates the conjugation between nanoparticles and the antibodies for Dimer detection.

## Methods

### Materials and Reagents

GoldMag (5 mg/mL) were provided by Xi’an GoldMag Nanobiotech Co., Ltd. (Xi’an, PRC). Boric acid, borax, Cetyltrimethylammonium bromide (CTAB), Poly(4-styrenesulfonic acid-co-maleic acid) (PSS-MA) (PSS-MA 3: 1, Mw ~ 20,000) were purchased from Sigma-Aldrich, USA. A pair of monoclonal D-dimer antibodies (antibody 1: M-2.1.16; antibody 2: M-1.2.57) were purchased from Roche. D-dimer were purchased from Meridian Chemicals, USA.

### Surface-Modified GoldMag with PSS-MA

The GoldMag were synthesized as described previously [17]. 13.3 mL of CTAB (50 mmol/L) was added to 13.3 mL of GoldMag (approximately 3 mg/mL). The mixture was mechanically stirred (200 rpm) combined with ultrasonication (SB-5200DTD, China) for 30 min followed by further stirring without ultrasonication for another 30 min. Particles were washed thoroughly with deionized water. The CTAB-GoldMag were re-dispersed in 10 mL of deionized water and 16 mL of 25% (*w*/*w*) PSS-MA solution was added followed by stirring (180 rpm) for 90 min. The modified particles were washed with deionized water twice and were dispersed in deionized water.

### Characterization

The PSS-MA-GoldMag were observed using transmission electron microscope (TEM, Hitachi H-600, Hitachi Corporation, Japan). The size distribution was analyzed by DLS (zeta-sizer, Malvern Instruments, UK). Fourier-transform infrared spectroscopy (FTIR, Thermo Nicolet 5700, Thermo Nicolet Corporation, USA) was used to characterize the functional groups of PSS-MA-GoldMag. A Metter Toledo SDTA 851e thermogravimetric analyzer (TGA) was used to analyze the proportion of polymer surface shell among the PSS-MA-GoldMag. The surface plasmon resonance (SPR) spectrum of GoldMag was recorded using a UV-2550 spectrophotometer (Shimadzu, Japan) in the wavelength range of 450–700 nm.

### Preparation of the Anti-D-dimer Antibody-PSS-MA-GoldMag Probe

One milligram of GoldMag was suspended in 1 mL borax/borate buffer (0.02 M, pH 7.4), containing 75 mg/mL of 1-ethyl-3-(3-dimethyl-laminopropyl) carbodiimide (EDC). Then, 100 μg anti-D-dimer antibody was added to the suspension followed by ultrasonication for 1 h. Three milliliters blocking buffer (0.02 M borax/borate buffer, pH 7.4, containing 5% BSA) were added into the mixture and were incubated for 1.5 h. After separation under an external magnetic field, anti-D-dimer antibody-PSS-MA-GoldMag composite was kept in borax/borate buffer under 4 °C. The conjugation of anti-D-dimer antibody on the PSS-MA-GoldMag surface was confirmed by SPR spectroscopy and dynamic light scattering (DLS) measurement.

The D-dimer solution was prepared by diluting a D-dimer stock solution (40 mg/mL) with calf serum. Three concentrations of D-dimer (0.6, 2, and 6 μg/mL) were used to optimize the reaction time between D-dimer and the anti-D-dimer antibody-PSS-MA-GoldMag complex. Ten microliters anti-D-dimer antibody-PSS-MA-GoldMag (0.3 mg/mL) were mixed with 80 μL of D-dimer solution (0.6, 2, and 6 μg/mL), and the mixture was incubated at 25 °C for 10, 20, 30, and 40 min. The wavelength of the SPR peak of the PSS-MA-GoldMag composite was read using a UV-2550 spectrophotometer.

D-dimer solutions with concentrations of 6, 3, 1.5, 0.75, and 0.3 μg/mL were prepared to analyze the relation between the concentration of D-dimer and the change of the SPR spectrum. Ten microliters of anti-D-dimer antibody-PSS-MA-GoldMag (0.3 mg/mL) were mixed with 80 μL of D-dimer solution (6, 3, 1.5, 0.75, and 0.3 μg/mL) and the mixture was incubated at 25 °C for 30 min. The wavelength of the SPR peak of PSS-MA-GoldMag was read using a UV-2550 spectrophotometer.

In order to evaluate if triglycerides, bilirubin, and hemoglobin can interfere with our protein detection system, D-dimer solutions (0 and 3 μg/mL) were prepared and 22 mg/mL triglycerides, 0.2 mg/mL bilirubin, and 2 mg/mL hemoglobin samples were added separately. Ten microliters of anti-D-dimer antibody-PSS-MA-GoldMag was added to the above mixtures, and the wavelength of the SPR peak of PSS-MA-GoldMag composite was read using a UV-2550 spectrophotometer.

## Results and Discussion

**Scheme 1:**
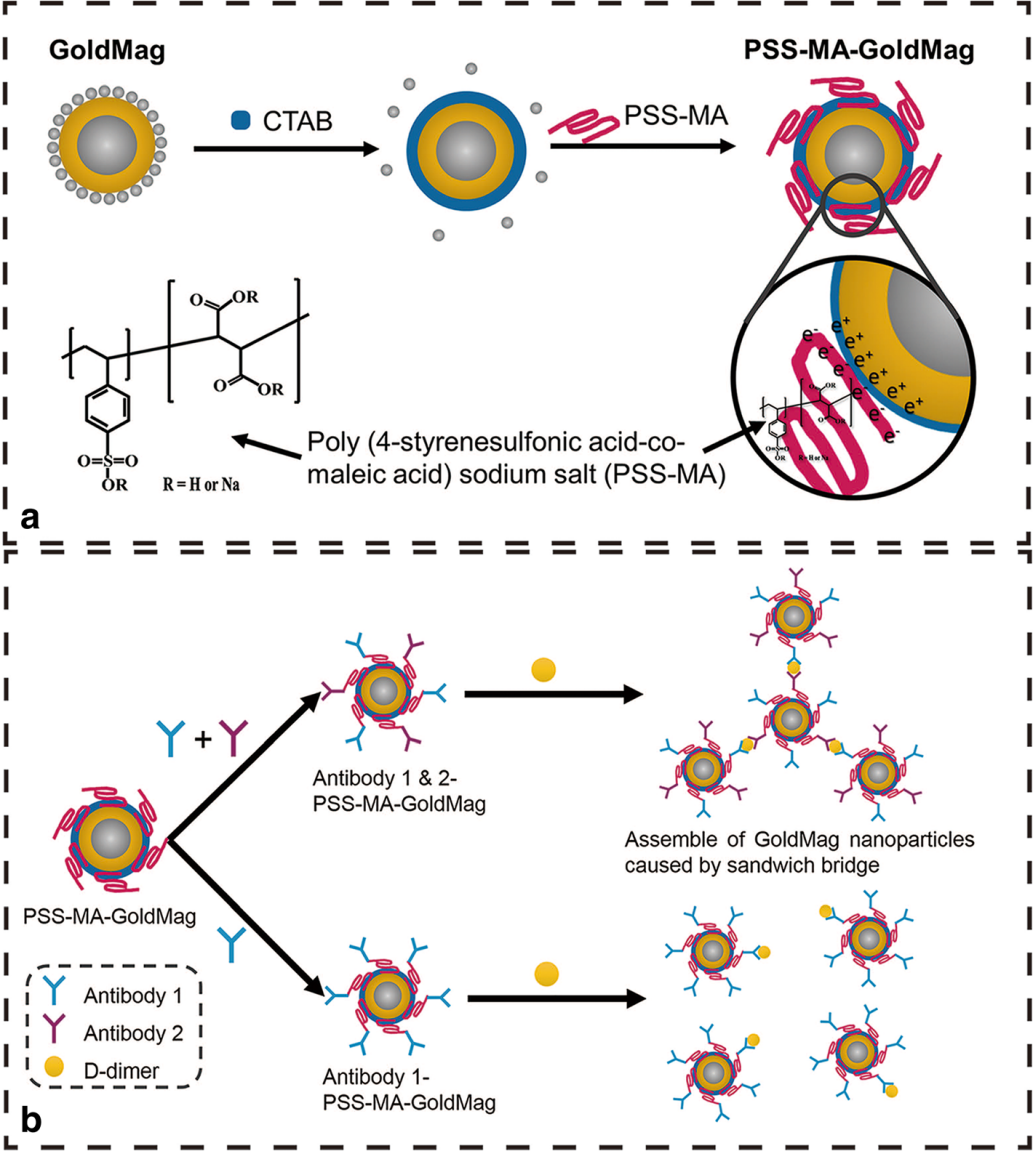
**a** The schematic illustration of the “over-all” process of surface modification of gold magnetic particles (GoldMag) and the molecular structure of poly(4-styrenesulfonic acid-co-maleic acid) (PSS-MA). **b** Schematic representation of the interaction of D-dimer with antibody 1 and 2-PSS-MA-GoldMag, as well as the interaction between D-dimer and antibody 1-PSS-MA-GoldMag

**Fig. 1:**
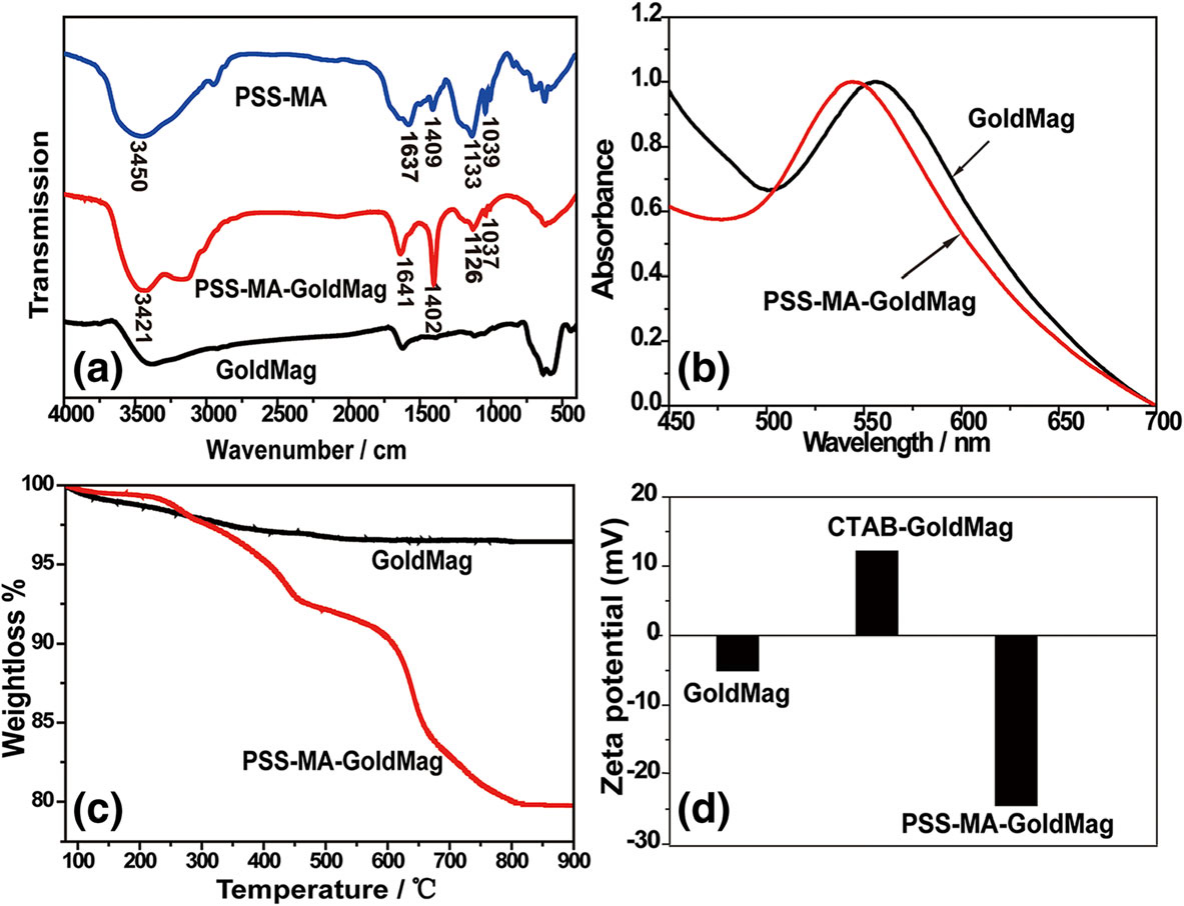
**a** FTIR spectroscopy of PSS-MA, PSS-MA-GoldMag, and GoldMag. **b** SPR spectroscopy of GoldMag and PSS-MA-GoldMag suspended in water. **c** TGA analysis of GoldMag and PSS-MA-GoldMag. **d** The change of zeta potential as a result of the modification

Scheme 1 illustrates the procedures developed for the surface modification and functionalization of particles for colorimetric detection of proteins. In order to study the adsorption and incorporation of PSS-MA on the PSS-MA-GoldMag structures, FTIR spectroscopic analyses, SPR spectroscopy, and TGA were performed. Figure 1a shows the FTIR spectrum for pure PSS-MA, PSS-MA-GoldMag, and GoldMag, respectively. The broad and strong band at 3000–3700 cm^−1^ corresponds to stretching of the –CO–OH groups and the hydroxyl groups of –SO_2_–OH in polymer chains [18]. Symmetric vibrations of sulfonate groups : (SO_3_^−^) were at 1037 and 1126 cm^−1^ and stretching vibrations of C = C of Benzene was at 1403 and 1637 cm^−1^ [19]. These characteristic absorption bands of PSS-MA were observed in PSS-MA-GoldMag demonstrating that the raw particles were successfully coated with PSS-MA. The change of surface chemical groups of the GoldMag was confirmed by a clear blue-shift of 11 nm from 555 to 544 nm in the SPR band after modification (Fig. 1b). The position and width of the SPR peak was related to the surface, the environment, and the dispersity of the nanoparticles [20–23]. TGA analysis indicated that the weight ratio of organic material to GoldMag was nearly 1:4 (Fig. 1c). At low temperature, weight loss can be attributed to dehydration; when the temperature rises, weight loss may be attributed to oxidative decomposition of the organic molecules on the surface of the modified particles [19, 24]. During the modification, CTAB-GoldMag were positively charged (+ 12.2 mV) and the particle surface became negatively charged (− 24.5 mV) after the PSS-MA coating on the particles surface (Fig. 1d). All above results show that PSS-MA are successfully adhered on the surface of nanoparticles.

**Fig. 2:**
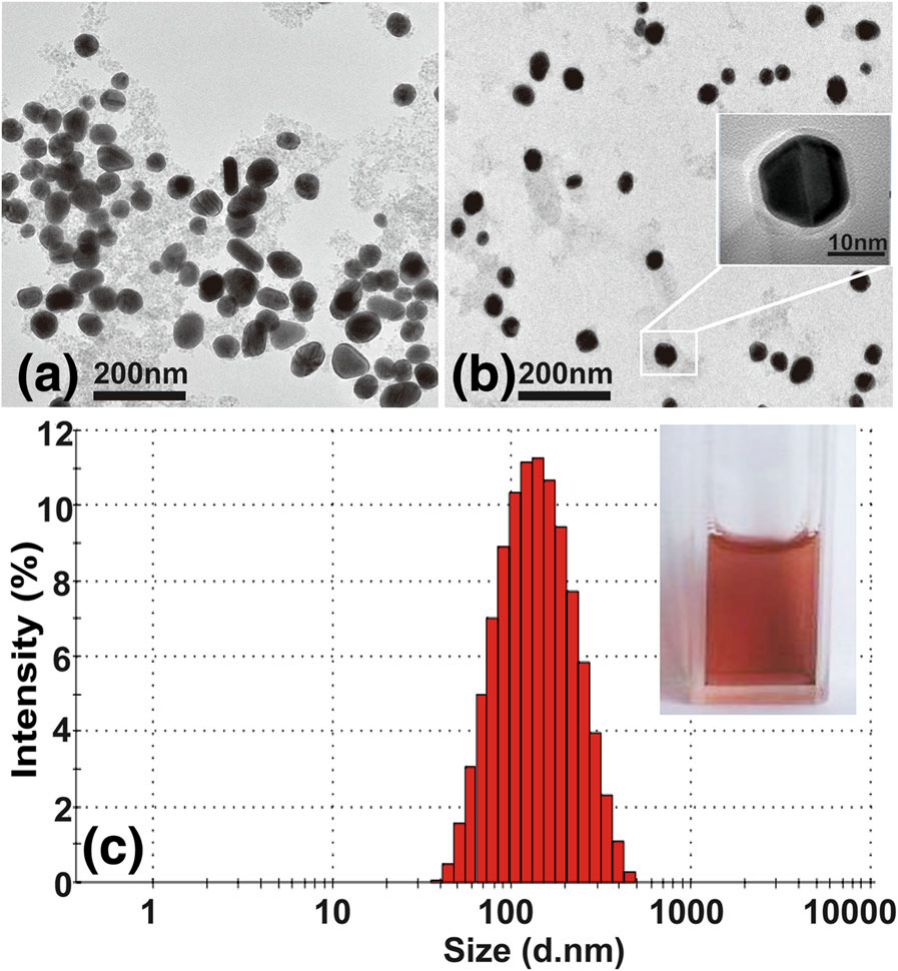
**a** TEM images of GoldMag. **b** TEM images of PSS-MA-GoldMag. The inset shows particles by polymer package. **c** Size distribution of GoldMag and PSS-MA-GoldMag. The inset shows the photograph of PSS-MA-GoldMag solution, which is showing a red color

The micrographs (Fig. 2a, b) of the GoldMag and PSS-MA-GoldMag obtained using TEM show that these particles were mono-disperse, after modification. The layer of polymer on the surface of the particles is clearly visible under the high-resolution electron microscopic image further indicating that the coating of polymer on GoldMag was successful (Fig. 2b). Figure 2c shows that the average diameter of PSS-MA-GoldMag is 116 nm. The PSS-MA-GoldMag suspension has a wine-red color which indicates a good stability of the nanoparticles (1 year in water).

**Fig. 3:**
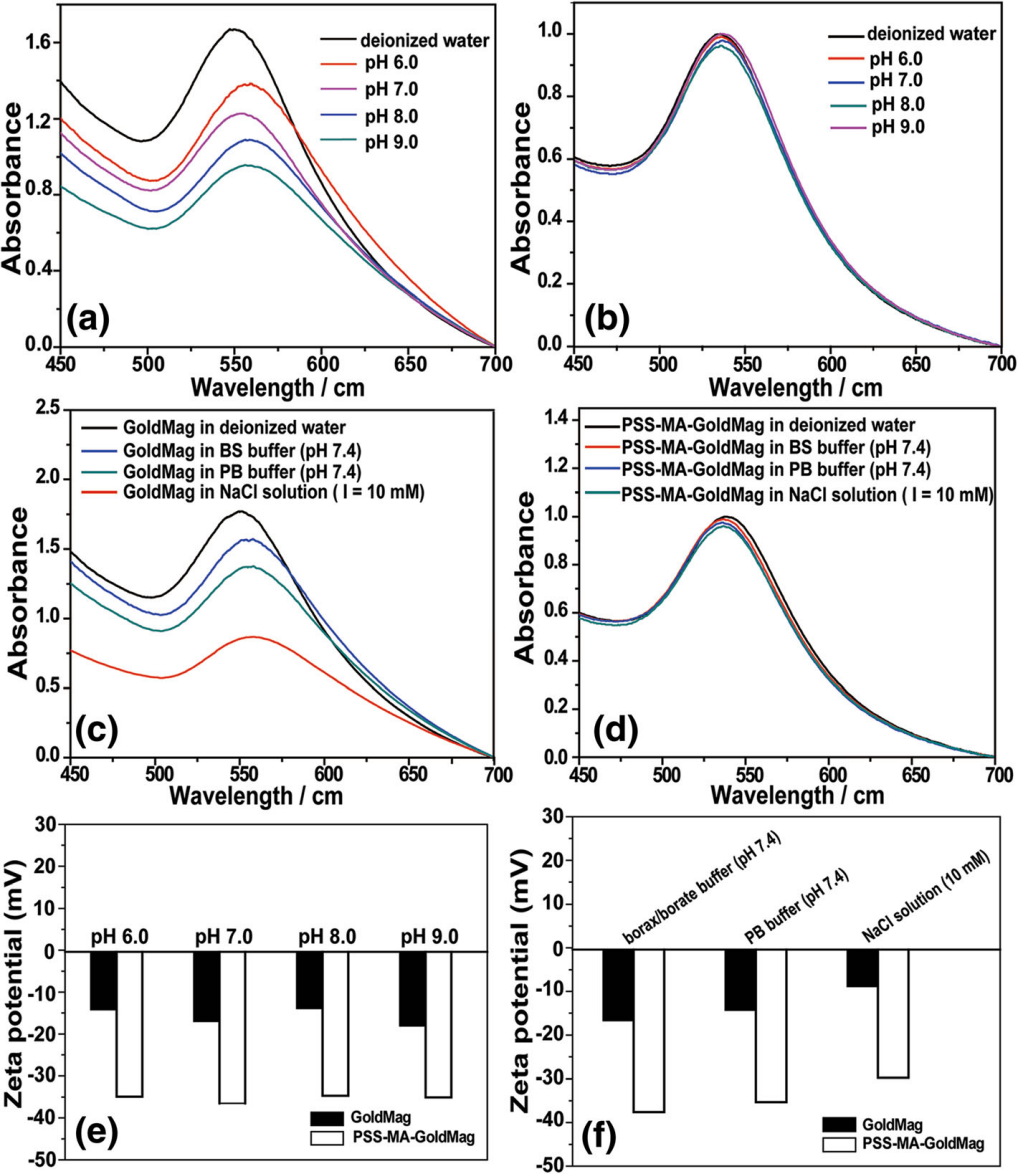
**a** The SPR spectra of GoldMag in borax/borate buffer (0.02 mol/L) with different pH values ranging from 6.0 to 9.0. **b** The SPR spectra of PSS-MA-GoldMag in borax/borate buffer (0.02 mol/L) with different pH values ranging from 6.0 to 9.0. **c** The SPR spectra of GoldMag suspended in borax/borate buffer (0.02 mol/L, pH 7.4), PB buffer (0.2 mol/L, pH 7.4) and in a electrolyte solution. **d** The SPR spectra of PSS-MA-GoldMag suspended in borax/borate buffer (0.02 mol/L, pH 7.4), PB buffer (0.2 mol/L, pH 7.4) and in electrolyte solution. **e** The change of zeta potential of GoldMag suspended in borax/borate buffer (0.02 mol/L) with different pH values ranging from 6.0 to 9.0. **f** The change of zeta potential of GoldMag suspended in borax/borate buffer (0.02 mol/L, pH 7.4), PB buffer (0.2 mol/L, pH 7.4), and in electrolyte solution

The stability of the optical characteristics of GoldMag and PSS-MA-GoldMag in buffer solutions with different pH values were analyzed by SPR spectroscopy [7]. As shown in Fig. 3a, the SPR characteristic peak was shifted to the longer wavelength from 555 nm when GoldMag were suspended in borax/borate buffer with a pH of 6.0 to 9.0. The composition of the buffer solution also influences the stability of the GoldMag suspension. The position of the SPR characteristic peak was moved to higher wavelengths (558 nm) when GoldMag were suspended in PB buffer (pH 7.4), borax/borate buffer (pH 7.4), and NaCl solution (10 mM) compared to those in deionized water (550 nm) (Fig. 3c). However, characteristic SPR peak (545 ± 2 nm) did not change significantly when PSS-MA-GoldMag were dispersed in electrolyte solution or in buffer solution with different pH values (Fig. 3b, d). Xia et al. and Storhoff et al. have also reported that aggregation of nanoparticles can make the SPR spectrum shift to longer wavelengths [25, 26]. The zeta potential of GoldMag and PSS-MA-GoldMag were also measured (Fig. 3e, f). It has been reported that a low zeta potential (less than ± 30 mV) will lead to particle agglomeration [27, 28]. When GoldMag were suspended in PB buffer (pH 7.4), borax/borate buffer (pH 7.4) and NaCl solution (10 mM), the zeta potential of GoldMag was − 16.7 ± 1.1 mV, − 14.3 ± 2.1 mV, and − 8.9 ± 1.5 mV, respectively. The zeta potential of GoldMag was − 14.2 ± 1.7 mV, − 17 ± 1.1 mV, 13.9 ± 1.7 mV, and − 18.1 ± 1.6 mV when particles were suspended in borax/borate buffer with pH of 6.0 to 9.0. However, the adherence of PSS-MA to GoldMag dramatically decreases its zeta potential in different electrolyte solution, as well as at different pH values. In all cases, the zeta potential was lower than − 30 mV. These results indicate that surface modification of GoldMag with PSS-MA significantly improves its resistance to changes of composition and of pH values of buffer solutions and assures a high level of dispersity [29].

**Fig. 4:**
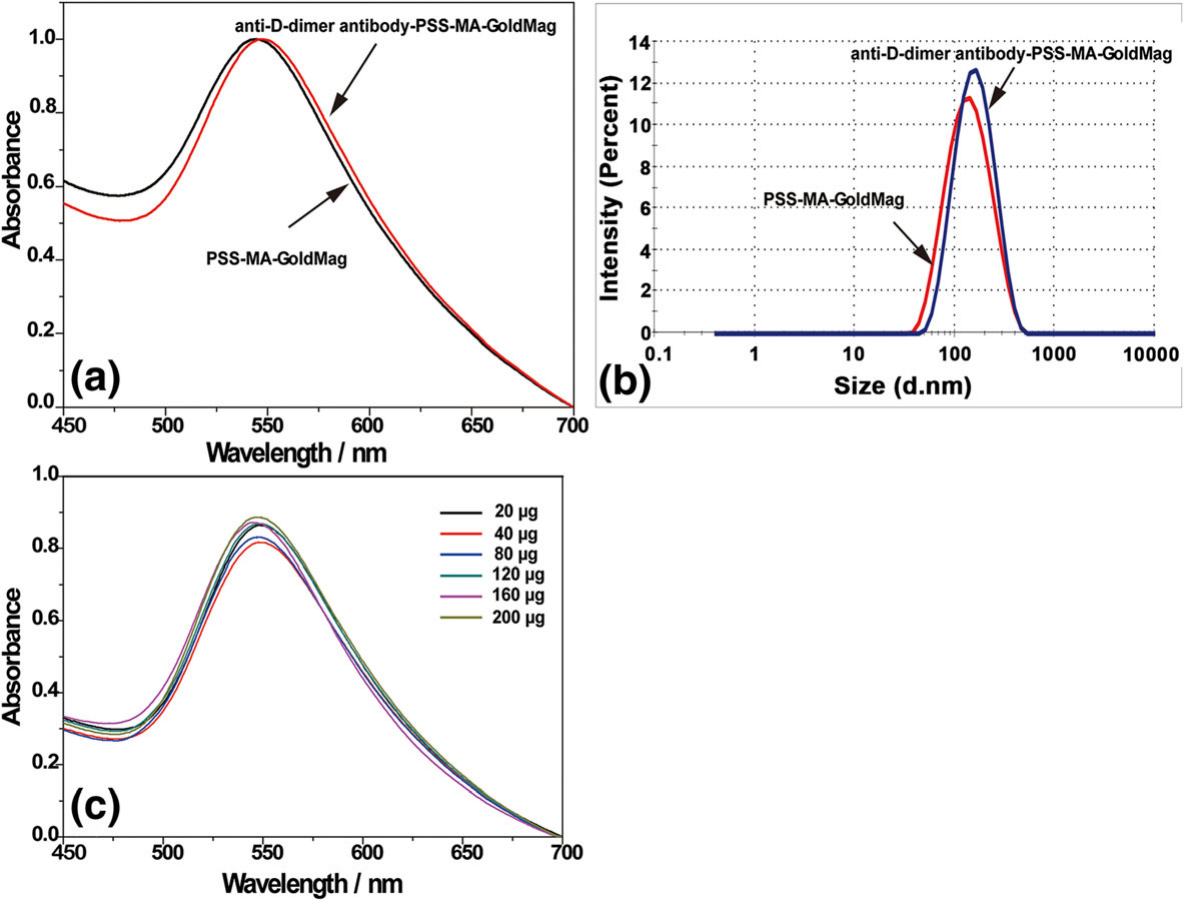
**a** The SPR spectra of PSS-MA-GoldMag and anti-D-dimer antibody-PSS-MA-GoldMag. **b** The size distribution of antibody-PSS-MA-GoldMag and PSS-MA-GoldMag. **c** The SPR peak of particles was not changed with increasing antibody concentrations

To investigate the interaction between protein and PSS-MA-GoldMag, anti-D-dimer antibodies were conjugated with PSS-MA-GoldMag through the reaction between protein amino groups and polymer’s carboxyl groups mediated by EDC. As shown in Fig. 4a, red-shift of the SPR peak was observed after the reaction of PSS-MA-GoldMag with anti-D-dimer antibodies, which indicates the conjugation of antibodies on nanoparticles [30, 31]. The increase of the average diameter of particles from 116 to 130 nm confirms the introduction of the anti-D-dimer antibody to the PSS-MA-GoldMag (Fig. 4b) [24]. The influence of antibody mass on the optical property of PSS-MA-GoldMag was evaluated through SPR spectroscopy after adding different amounts of anti-D-dimer antibody (from 20 to 200 μg) to the PSS-MA-GoldMag suspension. As shown in Fig. 4c, the SPR peak was not changed and was kept on 548 ± 3.0 nm, independent of the amount of antibody added.

**Fig. 5:**
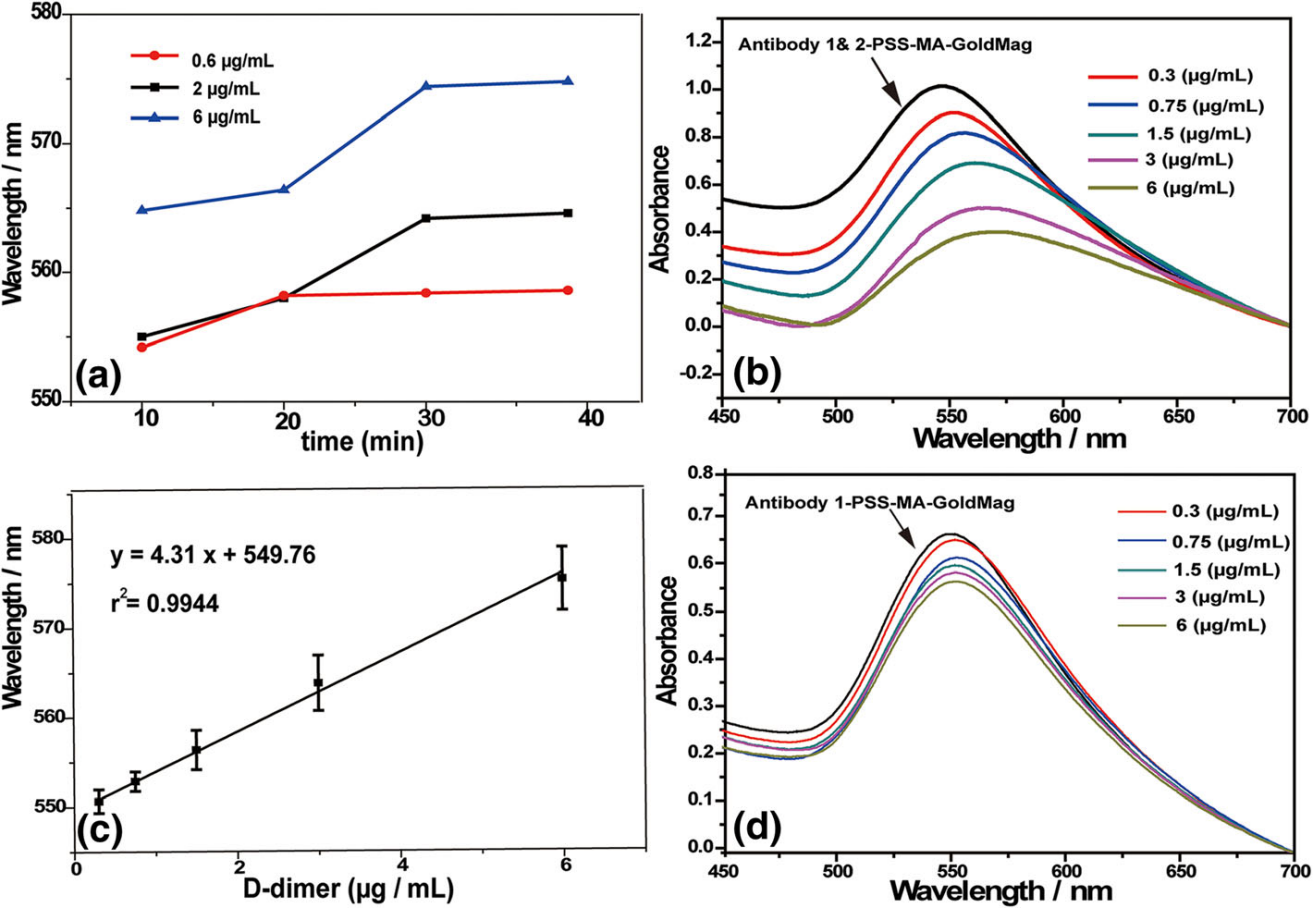
**a** The relationship between the reaction time and the SPR spectrum. **b** The redshift of SPR spectra due to aggregation of GoldMag particles caused by cross connection of D-dimer with probe A. **c** The curve plotted of the SPR peak of composites as a function of the D-dimer concentration. **d** The SPR spectroscopy of composites after addition of D-dimer to probe B

In order to evaluate the activity of the antibodies after conjugation on the PSS-MA-GoldMag, probe A and probe B were prepared by immobilizing a pair of antibodies (antibody 1 and antibody 2), and antibody 1 on PSS-MA-GoldMag, respectively. The interaction between the two types of probe and D-dimer was analyzed by SPR spectroscopy. The best reaction time was obtained by observing the change of the maximum of the SPR peak for 40 min after adding three concentrations of D-dimer (0.6, 2, and 6 μg/mL) to probe A. As shown in Fig. 5a, a significant shift in the surface plasmon band was observed resulted from the aggregation of PSS-MA-GoldMag through crosslinks between antigen-antibody sandwich bridge over time. At low concentration (0.6 μg/mL), there was no significant change on the SPR spectrum from 20 to 40 min, indicating that 20 min is enough for the reaction of 0.6 μg/mL or lower concentrations of D-dimer. However, using middle and high concentrations of D-dimer (2 and 6 μg/mL), the wavelength kept increasing 30 min. This indicates that the best reaction time is 30 min. As shown in Fig. 5b, the SPR spectrum of the composite particles revealed a distinct red shift in surface plasmon resonance from λ_max_ = 550 to 570 nm and intensity decrease of characteristic peak as the concentration of D-dimer increased from 0.3 to 6 μg/mL. This is provoked by the aggregation of PSS-MA-GoldMag caused by the cross connection of D-dimer with antibody 1 and antibody 2 on probe A. The aggregation of PSS-MA-GoldMag resulted in the change of the optical properties of the gold part of PSS-MA-GoldMag. Jiang et al. and Li et al. have also reported that aggregation of nanogold can make the SPR spectrum of nanoparticles red shift and border [32, 33]. However, no red-shift was observed in Fig. 5d because the interaction between D-dimer and antibody 1 was not able to lead to the assembly of nanoparticles. Furthermore, a distinguishable red-shift (5 nm) was observed even the amount of D-dimer added to probe A was as low as 0.3 μg/mL, which indicates that the limit of detection is below the diagnostic cut-off values (0.5 μg/mL) for this biomarker. A linear relation between the SPR spectral peak position and the D-dimer concentration was found in the range of 0.3–6 μg/mL (*r* ^2^ = 0.9944) (Fig. 5c).

**Fig. 6:**
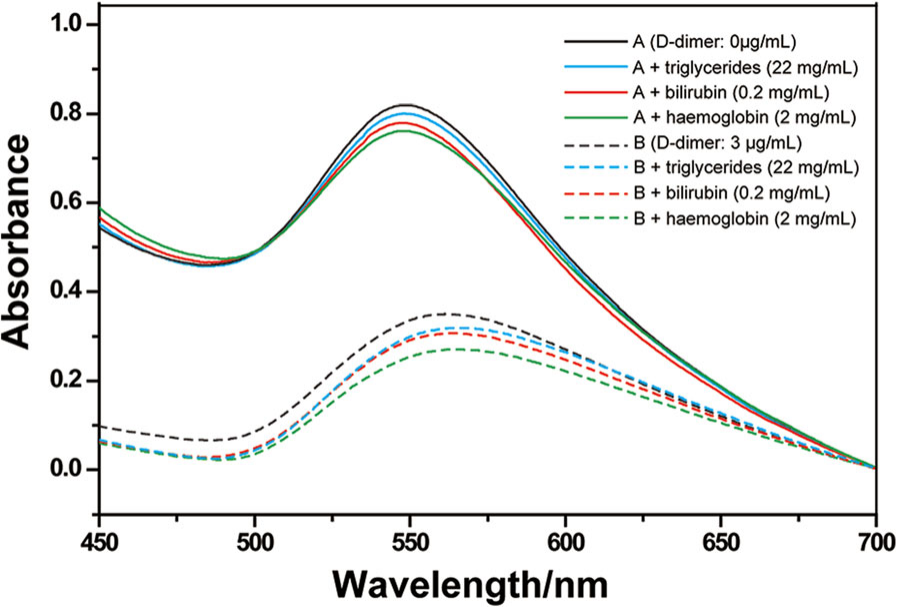
The SPR spectra of anti-D-dimer antibody-PSS-MA-GoldMag after reaction with D-dimer samples in the presence and absence of triglycerides, bilirubin, and hemoglobin

As shown in Fig. 6, no significant shift was found of the SPR characteristic peak of PSS-MA-GoldMag in the presence of triglycerides, bilirubin, and hemoglobin, respectively. This result indicates that our protein detection system has no interfering reactions with triglycerides, bilirubin, and hemoglobin.

These results show that PSS-MA-GoldMag is a promising magnetic nanoparticle for protein immobilization and can form a sandwich bridge on the particle surface via antibody 1-target protein-antibody 2. This makes PSS-MA-GoldMag valuable as a material for use in point-of-care optical detection of biomarkers.

In this study, PSS-MA was firstly used for modification of GoldMag (Scheme 1). The introduction of PSS-MA to the surface of GoldMag significantly improved its stability in buffer solution. In addition to the steric hindrance that is caused by the long polymer chain of PSS-MA, the carboxyl groups and the sulfo group in PSS-MA cause electrostatic repulsion between the nanoparticles [29]. This significantly enhances the stability of PSS-MA-GoldMag. The high number of carboxyl groups on the particle surface meets the requirement for conjugation with biomolecule.

## Conclusions

In the present study, an easy and rapid method for coating GoldMag with PSS-MA was reported. The results show that colloidal stability and dispersity of GoldMag were significantly improved through introduction of PSS-MA. The particles present good stability in buffer solution with a wide pH range. Moreover, the presence of carboxyl group offers the option of conjugating proteins to the nanoparticles. Taking D-dimer and its antibody as a model, it is found that the optical characteristic can be tailored through the crosslinking between antigen and antibody-nanoparticle composite. The results show that the PSS-MA modified GoldMag can serve as a very promising candidate for optical detection based on immunoassays.

## Abbreviations

DLS: Dynamic light scattering
FTIR: Fourier-transform infrared spectroscopy
GoldMag: Gold magnetic nanoparticles
MUA: 11-mercaptoundecanoic acid
PEI: Polyethylenimine
PSS: Polystyrenesulfonate
PSS-MA: Poly(4-styrenesulfonic acid-co-maleic acid)
SPR: Surface plasmon resonance
TEM: Transmission electron microscope
TGA: Thermogravimetric analyzer
